# Comparison of Geostatistical Interpolation and Remote Sensing Techniques for Estimating Long-Term Exposure to Ambient PM_2.5_ Concentrations across the Continental United States

**DOI:** 10.1289/ehp.1205006

**Published:** 2012-10-02

**Authors:** Seung-Jae Lee, Marc L. Serre, Aaron van Donkelaar, Randall V. Martin, Richard T. Burnett, Michael Jerrett

**Affiliations:** 1 Geospatial Development Department, Risk Management Solutions Inc., Newark, California, USA; 2 Department of Environmental Sciences and Engineering, School of Public Health, University of North Carolina at Chapel Hill, Chapel Hill, North Carolina, USA; 3 Department of Physics and Atmospheric Science, Dalhousie University, Halifax, Nova Scotia, Canada; 4 The Harvard-Smithsonian Center for Astrophysics, Cambridge, Massachusetts, USA; 5 Population Studies Division, Health Canada, Ottawa, Ontario, Canada; 6 Division of Environmental Health Sciences, School of Public Health, University of California, Berkeley, Berkeley, California, USA

**Keywords:** air pollution, chronic exposure, geostatistics, PM_2.5_, remote sensing

## Abstract

Background: A better understanding of the adverse health effects of chronic exposure to fine particulate matter (PM_2.5_) requires accurate estimates of PM_2.5_ variation at fine spatial scales. Remote sensing has emerged as an important means of estimating PM_2.5_ exposures, but relatively few studies have compared remote-sensing estimates to those derived from monitor-based data.

Objective: We evaluated and compared the predictive capabilities of remote sensing and geostatistical interpolation.

Methods: We developed a space–time geostatistical kriging model to predict PM_2.5_ over the continental United States and compared resulting predictions to estimates derived from satellite retrievals.

Results: The kriging estimate was more accurate for locations that were about 100 km from a monitoring station, whereas the remote sensing estimate was more accurate for locations that were > 100 km from a monitoring station. Based on this finding, we developed a hybrid map that combines the kriging and satellite-based PM_2.5_ estimates.

Conclusions: We found that for most of the populated areas of the continental United States, geostatistical interpolation produced more accurate estimates than remote sensing. The differences between the estimates resulting from the two methods, however, were relatively small. In areas with extensive monitoring networks, the interpolation may provide more accurate estimates, but in the many areas of the world without such monitoring, remote sensing can provide useful exposure estimates that perform nearly as well.

An extensive body of research has established the effects of exposure to fine particulate matter (PM_2.5_; particles < 2.5 μm in aerodynamic diameter) on morbidity and mortality (Hu 2009; Laden et al. 2006; Peters et al. 2001; Pope 2009). Studies using the American Cancer Society (ACS) cohort to assess the relation between particulate air pollution and mortality rank among the most influential and widely cited. Because of this robust association and a lack of other large cohort studies on long-term effects, the ACS studies have proven important to government regulatory interventions and health burden assessments (Pope et al. 2004). However, all of the national estimates from the ACS cohort have relied on central monitoring estimates of citywide PM concentrations, raising the possibility of substantial measurement error.

To better understand the adverse health effects of PM_2.5_-exposure, accurate estimates of the spatiotemporal variation of PM_2.5_ levels at fine space and time scales are needed. Although much of the PM_2.5_ variation is regional owing to the secondary formation of organic carbon, sulfates, and nitrates (Reiss et al. 2007), some PM_2.5_ mass is derived from local combustion, which may lead to variation at finer spatial scales. In some instances, these finer-scale variations in PM_2.5_ have been shown to associate with larger health effects than those that vary regionally (Jerrett et al. 2005), suggesting the potential importance of refining exposure predictions.

Predictions of particulate matter at a spatial scale finer than observation scales have been attempted several times recently using *a*) land use regression (LUR) models (Moore et al. 2007; Ross et al. 2007), *b*) generalized additive mixed models (Yanosky et al. 2008, 2009), *c*) hierarchical modeling (Sampson et al. 2011; Szpiro et al. 2010), *d*) geostatistical interpolation (Christakos and Serre 2000; Goovaerts et al. 2006; Liao et al. 2006), and *e*) remote sensing techniques (Liu et al. 2004; van Donkelaar et al. 2010). These approaches can be classified into two categories: those involving ground-level monitor–based estimation (*a*–*d* above), and those relying on satellite-based (monitor-free) estimation (*e*).

To date, only one study has systematically compared monitor-free estimation with an empirical monitor-based approach (Paciorek and Liu 2009). The study used empirical estimates from a Bayesian hierarchical model that employed land use information derived from a geographic information system (GIS). This carefully conducted analysis covering the eastern United States demonstrated that when land use and spatial correlations were incorporated into the estimation, little additional predictive value accrued from the satellite aerosol optical depth (AOD) retrievals. This insight was based on investigating the effect of the satellite retrievals on their PM_2.5_ estimation through cross-validation *R*^2^ and the corresponding mean squared prediction error. However, Kumar (2010) criticized the study for its inability to distinguish between natural and anthropocentric sources of PM_2.5_, which was in part due to uncontrolled meteorological influences.

A recent satellite-based study generated estimates of chronic PM_2.5_ exposure at 10-km gridded locations globally by integrating satellite-derived AOD and a chemical transport model that incorporates meteorology (van Donkelaar et al. 2010). These estimation surfaces depended on remotely sensed data collected during the period 2001–2006. The satellite-based estimates were, however, inevitably influenced by both random and systematic sources of uncertainty associated with AOD retrieval, varying relations between AOD and PM_2.5_, and temporal sampling biases (Hu 2009; Kumar 2010; Paciorek and Liu 2009).

The main goal of the present study was to compare estimates of long-term average PM_2.5_ for the continental United States based on a representative geostatistical kriging model (as a purely monitor-based approach using direct PM_2.5_ measurements) with estimates based on remote sensing (as a monitor-free approach). This approach contributes novel information to the literature by examining the entire continental United States rather than limiting the analysis to the eastern portions of the United States. In addition, our remote sensing model directly incorporates meteorological estimates into the calculation of PM_2.5_ concentrations. As mentioned above, Paciorek and Liu (2009) used a statistical model with auxiliary GIS data input, which is laborious and time-consuming to compile and execute. In contrast, we compared estimates based on remote sensing with those based on monitoring data only to determine the extent to which remote sensing improves estimation.

This study was part of a larger project designed to enhance the prediction capabilities of PM_2.5_ at finer spatial resolution over the United States and Canada and to conduct a detailed assessment of the health effects from particulate air pollution on all-cause and cause-specific mortality based on concentration–response functions from the ACS cohort.

## Methods

**Figure 1:**
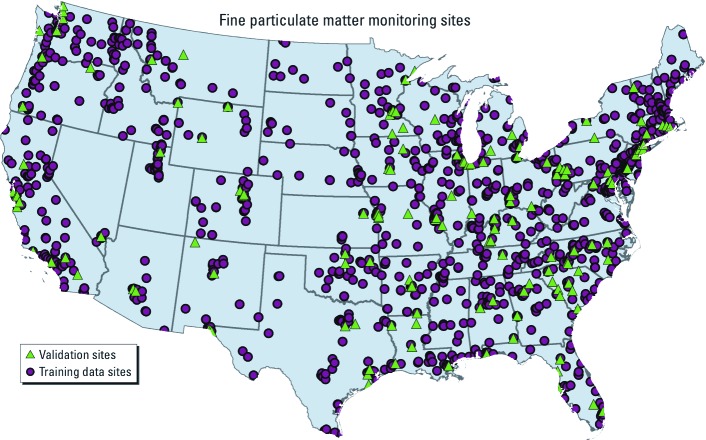
Monitoring stations for the U.S. EPA’s PM_2.5_ measurements. Training data for estimation were obtained from 1,315 sites. Data for validation were obtained from 147 randomly selected validation sites.

*Pollution data (monthly PM_2.5_ data).* We obtained daily PM_2.5_ measurements for the continental United States during 1997–2010 (1,742,020 monitor-days) from the Air Quality Subsystem (AQS) of the U.S. Environmental Protection Agency (U.S. EPA 2012). Our analysis was restricted to filter-based monitors using the Federal Reference Method (FRM), parameter code 88101 (U.S. EPA 2012). The initial daily data were aggregated to obtain monthly averages reflecting seasonal PM_2.5_ variation (Bell et al. 2007), which reduces the computational burden associated with the use of daily measurement data. Because many monitoring stations were in service for only part of the reporting time period, we included only monitoring stations with ≥ 50% of possible complete samples in a month. Although the U.S. EPA does not provide monthly PM_2.5_ averages on its air pollution data center web sites, quality assessment was conducted by comparing arithmetic yearly averages based on the monthly data against annual averages for FRM monitors available from the U.S. EPA (2012). The correspondence between U.S. EPA annual averages and annual averages based on the monthly averages was very strong (*r* = 0.996). Monthly values were retained for modeling if the data were determined to have ≥ 50% completeness, resulting in monthly data from 1,462 sites for the interpolation method. We selected a random sample of 147 of these sites (Figure 1) for the validation study described below.

*Satellite-based PM_2.5_ estimates of long-term average (2001–2006).* We obtained 6-year average PM_2.5_ estimates that were derived for a previous study using an integrated remote sensing–chemical transport model approach (van Donkelaar et al. 2010). Ground-level concentrations of PM_2.5_ were estimated using satellite atmospheric composition data combined with local coincident scaling factors from the GEOS-Chem chemical transport model (GEOS-Chem 2012). Specifically, AOD data from the MODIS (Moderate Resolution Imaging Spectroradiometer) and MISR (Multiangle Imaging Spectroradiometer) satellites were regridded to a 0.1 × 0.1° resolution (about 10 × 10 km). The AOD retrievals were translated into estimated ground-level PM_2.5_ using the output from GEOS-Chem simulations. As part of their analysis, van Donkelaar et al. (2010) removed any AOD with an anticipated bias of > 0.1 or 20% (whichever was larger), and limited the analysis to spatial points with ≥ 50 acceptable-quality near-daily AOD values. van Donkelaar et al. (2010) estimated 6-year average exposures in part because satellite information was missing for many spatial points of the 10-km remote sensing grid over time: Averaging data over a 6-year period resulted in comprehensive spatial coverage of the satellite AOD data (about 95% global coverage), which was used to derive long-term PM_2.5_ exposure estimates for the 10-km gridded locations. For the present study, we used this estimation surface, hereafter referred to as RS, as a baseline method for comparison with 6-year average monitor-based PM_2.5_ exposure estimates derived from ground-level measurements for the same period.

Both monitor-based (measured) and monitor-free (satellite-based) PM_2.5_ were initially linked to longitude and latitude (in degrees) but were thereafter projected to a planar surface (in kilometers) for our analysis.

*Kriging.* Kriging is a generalized linear regression technique that accounts for spatiotemporal correlations between samples and provides optimal estimates at unmonitored points. The optimal estimates may be obtained by finding weights that minimize the mean square error (Olea 1999). Many linear kriging methods do not integrate information from physical models or higher-order statistics regarding nonlinearity, non-Gaussianity, or data uncertainty, but kriging is still a useful method to interpolate numerous space/time dynamics. In our analysis, we used simple kriging (SK) with a refined smoothing filter [referred to as the composite space/time mean trend model (CSTM)] as described below.

The space/time random field (S/TRF) *Z*(***p***) = *Z*(***s****,t*) (Christakos 2000) is a random variable (i.e., the PM_2.5_ distribution over space and time) indexed by the two-dimensional spatial location *s* and the one-dimensional temporal point *t*, and *Y*(***p***) = log[*Z*(***p***)] as its log transformation. *m_Y_*(***p***) is a deterministic function representing the global mean trends in *Y*(***p***) constructed such that the deterministic transformation *X*(***p***) = *Y*(***p***) – *m_Y_*(***p***) produces a homogeneous stationary S/TRF defined by a locally constant mean *m_X_*(***p***) = E[*X*(***p***)], where E[.] is the expectation operator, and by a covariance *c_X_*(***p***, ***p***´) = E{[*X*(***p***) – *m_X_*(***p***)][*X*(***p***´)-*m_X_*(***p***´)]} that is a function of the spatial lag *r* = ||***s*** *–* ***s***´|| and temporal lag τ = |*t – t*´| between points ***p*** = (***s***,*t*), and ***p***´ = (***s***´,*t*´). The mean trend function characterizes the systematic trends and spatiotemporal structures of the PM_2.5_ distribution, whereas the covariance function addresses the correlation structures for the S/TRF, taken at a pair of points.

The SK estimation ^^^χ*_k_* of *X*(***p***) at estimation points *k* is a linear combination of measurements χ_d_ [i.e., the realization of *X*(***p***) at data points ***p***_d_] given by

^^^χ*_k_* = *m_X_*(***p***_k_) + λ^Τ^[χ_d_ – ***m****_X_*(***p***_d_)], [1]

where λ is a column vector of SK weights (in general, the closer the composite space/time separation between ***p***_k_ and ***p***_d_, the greater the weight), *m_X_*(***p***_k_) is the mean trend of *X*(***p***) at the estimation point ***p***_k_, and ***m****_X_*(***p***_d_) is a column vector of expected values for *X*(***p***) at the data points. The vector of SK weights was given by (Olea 1999),

λ^T^ = ***c****_k,d_****c****_d,d_*^–1^, [2]

where ***c****_k,d_* = ***c****_X_*(***p***_k_,***p***_d_) is a row vector of covariance for *X*(***p***) between the estimation point and data points, and ***c****_d,d_* = ***c****_X_*(***p***_d_,***p***_d_) is a covariance matrix for *X*(***p***) between the data points. Equations 1 and 2 are based on the so-called ordinary S/TRF that is a limiting case of a more generalized S/TRF, accounting for spatial nonhomogeneity and temporal nonisotropy (Christakos 1992).

We implemented space/time SK estimation using the geostatistical library function *BMElib* written in MATLAB (BMElib 2012; Christakos et al. 2002). *BMElib* provides an extensive suite of computational functions with which to model the space/time global trend *m_Y_*(***p***) and space/time residual covariance *c_X_*(***p***, ***p***´) functions [see Supplemental Material, Equation S1 (http://dx.doi.org/10.1289/ehp.1205006)] and to derive kriging estimates.

*Global mean trend models.* One way to obtain the global mean trend *m_Y_*(***p***) = *m_Y_*(***s****,t*) is to use the separable space/time mean trend model (SSTM) (Christakos et al. 2002). The SSTM approach first calculates raw spatial means by averaging the measurements at fixed monitoring sites, and raw temporal means by averaging the measurements at fixed monitoring time events. Next, an exponential filter is applied to the raw spatial and temporal means to derive the smoothed spatial mean component *m_Y_*(***s***) and smoothed temporal component *m_Y_*(*t*), respectively. For example, the smoothed *m_Y_*(***s***) value is calculated for any spatial point ***s*** of interest as the weighted average of the raw spatial means, where the weights decrease exponentially with the distance between each ***s*** and the location of the monitoring station where that raw spatial mean was calculated. The space/time mean trend, *m_Y_*(***s***,*t*) is combined as an additive function of *m_Y_*(***s***) and *m_Y_*(*t*), that is,

*m_Y_*(***s***,*t*) = *m_Y_*(***s***) + *m_Y_*(*t*) – μ, [3]

where μ is the mean value of *m_Y_*(*t*), such that *m_Y_*(*t*) – μ represents the fluctuation of *m_Y_*(*t*) around its mean. The *m_Y_*(***s***) denotes persistent spatial characteristics in PM_2.5_, whereas the *m_Y_*(*t*) captures seasonal trends in PM_2.5_. The mean trend model is “separable” because each of the smoothed components relies on either a purely spatial or purely temporal metric. The SSTM has performed well in numerous smaller-scale (i.e., state- or citywide) geostatistical studies (Christakos and Serre 2000; Lee et al. 2010, 2011).

**Figure 2:**
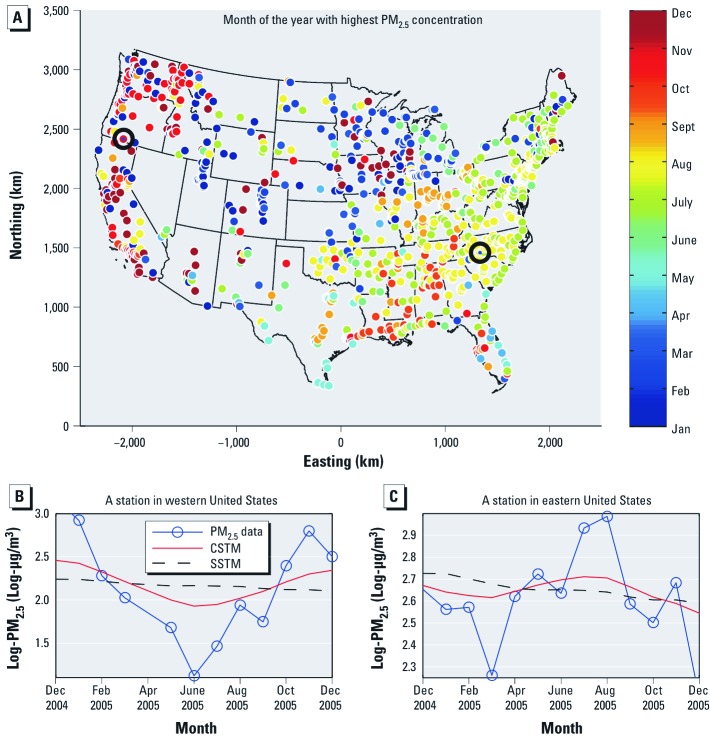
Periodicity shift in time across the United States and mean trend models fitting the shift. Map of the United States indicating the month of the year when the monthly average PM_2.5_ concentration was highest (*A*); circles indicate individual monitoring sites. PM_2.5_ measurements and corresponding CSTM and SSTM for a single monitoring site in the western (*B*) and in the eastern United States (*C*); the sites are indicated by black circles in (*A*)*.*

A visual inspection of the time–series of PM_2.5_ plotted for all monitoring stations (not shown) revealed a 1-year temporal periodicity in PM_2.5_ levels due to seasonal effects. This periodicity shifted in time, depending on where the monitoring station was located. For example, PM_2.5_ levels at one monitoring station in the western United States (Figure 2A,B) were highest in November, whereas the PM_2.5_ levels at another station in the eastern United States (Figure 2A,C) were highest in August. We assumed the periodicity could be fit using weights calculated based on an exponentially decaying function. The CSTM (Figure 2B,C) is based on composite space/time metrics (neither purely spatial nor purely temporal) and applies an exponential spatial-averaging to the selected measurements to obtain a smoothed mean trend value for each spatiotemporal coordinate ***p***_j_ = [***s***_j_ *t*_j_],


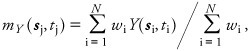
[4]

where *Y*(***s***_i_,*t*_i_) is the log-PM_2.5_ measurement at point ***p***_i_ = [***s***_i_,*t*_i_] such that the Euclidean distance between ***s***_i_ and ***s***_j_, *d*(***s***_i_, ***s***_j_) is **≤** 100 km, |*t*_i_ – *t*_j_| is **≤** 12 months, and the weight *w*_i_ is equal to *exp*[–*d*(***s***_i_, ***s***_j_)/*a*_r_ – |*t*_i_–*t*_j_|/*a*_t_], where *a*_r_ and *a*_t_ are respectively the spatial and temporal ranges of the exponential smoothing function (in our example *a*_r_ = 50 km and *a*_t_ = 3 months).

We used a cross-validation procedure to compare the accuracy of kriging PM_2.5_ estimates based on the CSTM (referred to as KC hereafter) versus the SSTM (KS) as described in detail in Supplemental Material, pp. 2–3 (http://dx.doi.org/10.1289/ehp.1205006). Because the CSTM-based estimates outperformed the SSTM-based estimates, we used the CSTM to derive our global mean trend *m_Y_*(***p***) and KC for comparison with RS, respectively.

*Validation of KC with RS.* Because RS corresponded to chronic exposures equivalent to 6-year PM_2.5_ average values, we compared it to the 6-year average of monthly KC. For validation purposes, we removed all 6-year PM_2.5_ averages measured at the 147 randomly selected validation monitors, and derived KC based on data from the remaining 1,315 training monitors only. Next, we derived KC and RS of the 6-year PM_2.5_ averages for each of the validation monitors and compared them with the removed measured (true) values to quantify the mapping accuracy of KC and RS. Finally, we investigated how mapping accuracy changes for each validation monitor as a function of the distance with its closest neighbor among the training monitors. Details of this validation procedure are as follows:

We selected one validation site from which to extract monthly measurements between January 2001 and December 2006 and defined them as a vector of validation values χ_v_.
We used the kriging equation (Equation 1) with the training data set to obtain monthly KC χ_k_ of PM_2.5_ at the validation monitor (so that each value of the vector χ_k_ is a set of estimates (KC) of the corresponding vector χ_v_).
We calculated the 6-year averages based on the monthly values (i.e., the average ^^^χ_v_ of the χ_v_ values and the average ^^^χ_KC_ of the χ_k_ values).
We extracted RS ^^^χ_RS_ (remote sensing-based estimation surface averaged over the period 2001–2006) for that validation monitor.
We repeated the steps above, choosing another validation site among the 147 sites.
Of the 147 sets of ^^^χ_v_, ^^^χ_KC_, and ^^^χ_RS_ values, we retained 74 with data that were ≥ 80% complete (i.e., ≥ 58 of 72 possible monthly records during 2001–2006). This 80% completeness criterion may be customized, but we found that selecting any percent > 80 did not substantially alter the validation results (data not shown).
We categorized the 74 validation sites into six groups based on the spatial lag between the validation monitor and its closest training monitor. The length for the first three classes was equidistant (15 km), and thus the upper limit of the third class was about 45 km. This left the other 15 validation sites to assign: The length of the last three classes was reduced to 10 km (fourth lag, 50–60 km; fifth lag, 60–70 km; and sixth lag, > 70 km, although the minimum value for the last class was actually 90.8 km) to assign a relatively equal number of the remaining validation sites to each of the last three classes.
For each spatial lag class *l* = 1,…,6 and for each estimation *m* = KC or RS, we calculated the mean error [ME*_m_*^(^*^l^*^)^], the mean square error [MSE*_m_*^(^*^l^*^)^] and the mean absolute error [MAE*_m_*^(^*^l^*^)^] (Lee and Wentz 2008). For each spatial lag class we calculated the percent change in MSE and MAE from RS to KC [e.g., % change in MSE^(^*^l^*^)^ = {[MSE_KC_^(^*^l^*^)^ – MSE_RS_^(^*^l^*^)^]/MSE_RS_^(^*^l^*^)^} × 100, so that a negative percent change means that KC has a lower estimation error than RS]. We also calculated the correlation coefficients (i.e., Pearson’s *r* and Spearman’s ρ) between the validation values ^^^χ_v_ and corresponding estimates ^^^χ_KC_ and ^^^χ_RS_ within that class lag.

This procedure produced a validation that goes beyond the traditional approach of examining the accuracy of the method across the entire domain with an MSE. Instead, we examined the distance away from a monitor at which each method produces a more accurate result.

## Results

*KC vs. RS.* As evidenced by its lower MSE and MAE statistics and negative values for percent change in MSE and MAE (Table 1), KC outperformed RS consistently up to the fifth spatial lag class (corresponding to an average distance of 65.5 km from the estimation point to its nearest measurement site), but conversely RS became more accurate when the estimation point was about 106 km from its nearest measurement site. The estimation accuracy of a method along the spatial lags may be affected by *a*) spatial distance (between estimation and data points), and *b*) data quality (whether estimates are based on PM_2.5_ measurements or on auxiliary information such as AOD). In the absence of nearby measurements, RS based on local AOD was more accurate than KC based on measurements at a distant monitor.

**Table 1:** Validation statistics for the KC and RS methods.

No. of validation monitors	32	13	14	4	6	5
Mean distance from monitors to estimation sites (km)	7.6	20.9	39.1	56.5	65.5	106.0
MSE
KC	1.229	1.610	1.871	0.699	1.145	2.762
RS	4.516	5.307	7.320	1.555	3.014	2.230
MSE change (%) RS to KC	–72.796	–69.672	–74.438	–55.066	–62.017	19.270
MAE
KC	0.799	1.084	1.172	0.781	0.993	1.377
RS	1.551	1.883	2.264	1.019	1.626	1.279
MAE change (%) RS to KC	–48.512	–42.401	–48.261	–23.384	–38.940	7.223
ME
KC	0.228	0.035	0.511	0.108	–0.586	–0.842
RS	0.202	0.402	2.264	1.019	0.148	0.088
Pearson r
KC	0.929	0.873	0.882	0.071	0.861	0.886
RS	0.733	0.534	0.879	0.644	0.447	0.908
Spearman ρ
KC	0.826	0.786	0.917	0.200	0.600	0.800
RS	0.546	0.615	0.943	0.400	0.600	0.900


The MSE and MAE percent change from RS to KC varied gradually across classes. For example, the MSE percent change varied gradually from –72.80% in the first class to –55.07% in the fourth class (as opposed to the unstable variation in the absolute value MSE for KC, which went from 1.229 in the first class to 0.699 in the fourth class) (Table 1). Table 1 also shows that the MSE/MAE percent changes were negative from the first to the fifth classes, but the changes are positive at the sixth class, indicating that KC perfomed better in the first five classes (with shorter spatial lags), whereas RS perfomed better in the sixth class (with the longest spatial lag).

KC was positively and strongly correlated with corresponding measurements at any class, as indicated by the *r* values close to 1 (Table 1), with the exception of the *r* value of 0.071 at the fourth class, which we attribute to a small sample size and the presence of an outlier, which once removed resulted in a recalculated Pearson’s *r* of 0.930. Apart from that outlier, KC *r* values were better (closer to 1) than those of RS for the first five classes, whereas the opposite was true for the sixth class. The Spearman’s ρ values revealed a similar pattern, also indicating that KC performs better for short spatial lags, whereas RS becomes more accurate at longer distances.

**Figure 3:**
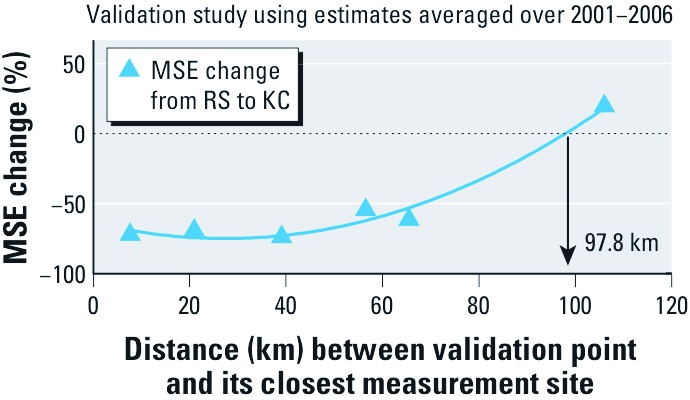
Percent change in MSE from RS to KC shown as a function of the distance between the validation point and its closest measurement site. The curve indicates a second order polynomial regression model that fits the MSE changes.

To elucidate the spatial lag at which RS becomes more accurate, we plotted the MSE percent change as a function of the spatial lag using the second order polynomial regression that fit the MSE changes in a least-squares sense (*R*^2^ = 0.9736) (Figure 3). The MSE percent change was negative (i.e., KC performed better than RS) for separation distances of < 97.8 km, whereas the opposite was true beyond that separation distance. However, a different validation data set or classification may have yielded different results for the specific distance at which RS performed better than KC.

*Implication to mapping.* We generated 10-km gridded estimation points over space by calculating a weighted average of KC and RS. The weights were negatively related to the MSE of KC and RS, which varied as a function of the spatial distance between the estimation point and the closest monitoring site. KC and RS were equally weighted when the separation distance was 97.8 km, the distance at which MSE for KC and RS were equal. For a location < 97.8 km from a monitoring site, we set KC MSE (MSE_KC_) to 1, and calculated a relative MSE for RS (MSE_RS_) = 100/(100+*q*), where *q* was the negative percent change in MSE_RS_ relative to MSE_KC_ (Figure 3). KC and RS weights were respectively defined by

MSE_RS_ /(MSE_RS_ + MSE_KC_), [5]

MSE_KC_ /(MSE_RS_ + MSE_KC_). [6]

For any *q* ≤ –100, the contribution of RS would be negligible, and we set the KC and RS weights to 1 and 0, respectively. For a location ≥ 97.8 km from a monitoring site, we set the MSE_RS_ to 1, and calculated the MSE_KC_ = 100/(100-*q*), where q was a positive value. KC and RS weights were also based on Equations 5 and 6, but they were respectively set to 0 and 1 for any *q* ≥ 100.

**Figure 4:**
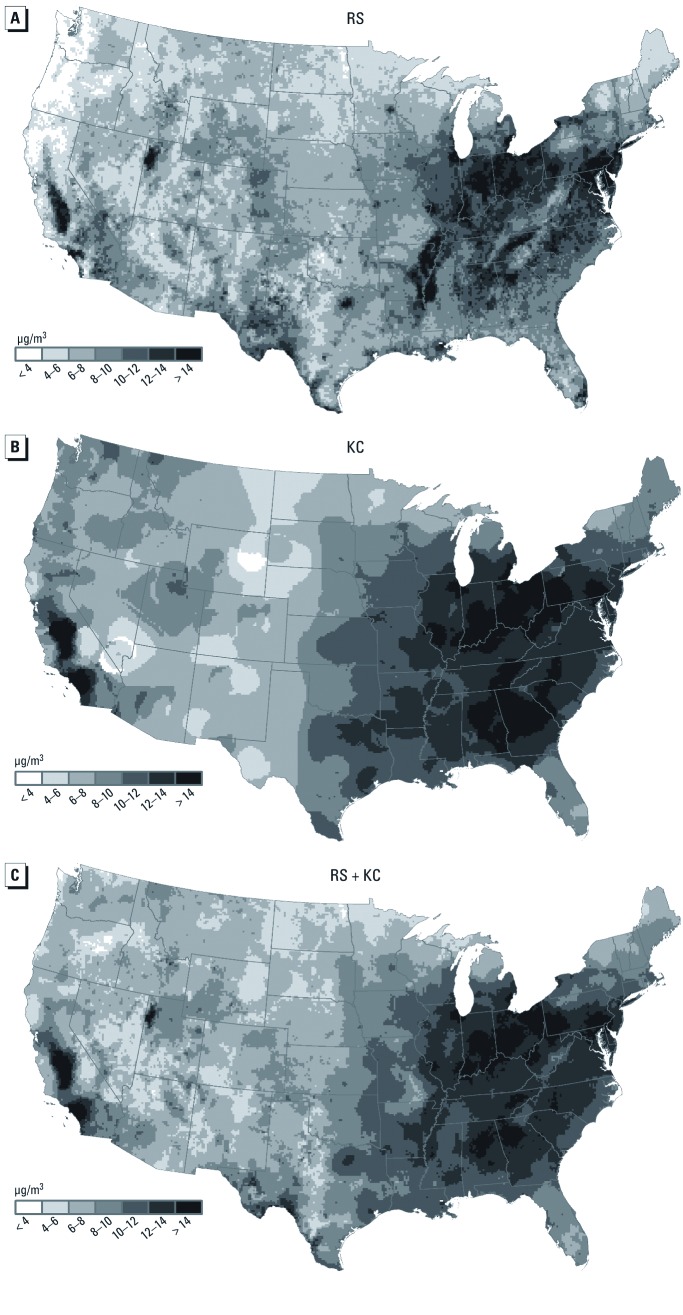
Average PM_2.5_ exposure estimates at 10-km gridded locations for 2001–2006 based on (*A*) RS (integrated remote sensing-meteorology model), (*B*) KC (monitor-based model), and (*C*) a combination of RS and KC.

We generated 10-km gridded estimation points over space (the grid cell size used by RS) using RS (Figure 4A) and KC (Figure 4B) to calculate a weighted average based on both approaches. The resulting map (Figure 4C) shows estimated PM_2.5_ levels that were higher than those based only on KC in areas with sparse monitoring data in which monitor-based KC may not be accurate.

## Discussion

We compared the best available long-term PM_2.5_ estimates using monitor-based (KC) and monitor-free (RS) methods. The multiyear duration (2001–2006) we depended on may be interchangeable with the time duration of 2–5 years commonly found in long-term PM exposure–health effect studies. We found a cutoff separation distance of 97.8 km at which the two methods showed an identical estimation performance. PM_2.5_ measurements contributed significantly to the estimation of ≤ 97.8 km from the measurement site, but the contribution of the measurements to the estimation was negligible beyond that spatial range. Based on the validation results, KC was preferable for estimating chronic exposure to PM_2.5_ up to about 100 km from a measurement site, whereas RS performed better beyond that distance.

We used a weighted average to combine KC and RS according to the distance between the measurement point and estimation site, but may refine this approach in the future by, for example, including more monitors from the 1,315 training sites for validation purposes (although the complete set of 72 monthly records for 2001–2006 were available from only 160 of the monitors). Moreover, the KC using ground-level measurements is potentially more accurate than the RS for deriving estimates over shorter time scales (e.g., yearly or monthly). Therefore, developing an efficient way to combine information from RS and ground-level measurements (e.g., using the correlation structures between indirect RS and direct ground-level measurements rather than simply taking a weighted average of collocated KC and RS) may lead to substantial improvements in the estimation of PM_2.5_ exposure at space/time resolutions of biological relevance for health studies.

The KC we developed may be useful for a wide variety of human health studies, but RS appears to perform better for estimating exposures of populations that live at relatively greater distances from monitors. A significant portion of the U.S. population (according to statistics from the U.S. Census Bureau for 2000) resides near monitors [i.e., 74.2% were within 25 km of a monitor, 89.8% were within 50 km, 96.5% were within 75 km, and 98.5% were within 97.8 km (U.S. Census Bureau 2012), the cut-off distance beyond which RS was more accurate in our analysis]. Therefore, in jurisdictions with fairly dense monitoring networks such as the United States, it may be appropriate to assess exposure with KC using nearby ground-level measurements. However, in most other regions of the world where few PM_2.5_ monitors exist, RS provides a critical information source (Brauer et al. 2011). Because the differences between estimates based on the two methods were relatively small, for the many areas of the world without dense monitoring, remote sensing can provide useful exposure estimates that perform nearly as well as ground-level–based estimates from a dense network.

The ability to estimate PM_2.5_ based on satellite remote sensing of AOD has advanced rapidly in recent years (Hoff and Christopher 2009). Further improvements in accuracy can be expected through advances in retrieval algorithms to infer AOD from measured radiation, in improved calculation of the AOD to PM_2.5_ ratio, and in new satellite instrumentation. As PM_2.5_ estimation based on remote sensing continues to improve, RS may outperform KC at distances that are closer to monitor locations than the current 97.8-km cut point identified in our analysis. First, additional information concerning land use, traffic, and population may be incorporated to inform PM_2.5_ concentration estimates (Paciorek and Liu 2009). A multivariate interpolation framework can process the additional data. Second, it may be possible to use chemical models to derive a more informative covariance structure and thus more accurate interpolation estimates, particularly when PM_2.5_ monitor data are limited. Development of techniques to combine information from remote sensing, models, and monitors should ultimately yield the best estimate of PM_2.5_ distribution.

## Conclusions

We developed a geostatistical interpolation method to estimate chronic exposure to PM_2.5_ over the United States, and compared these monitor-based estimates with monitor-free RS for constructing an improved assessment of long-term PM_2.5_ exposure. We identified the distance of 97.8 km between estimation sites and monitors within which KC was more accurate than RS, and conversely beyond which RS was superior. This cut-off radius may be used to combine KC and RS to build an up-to-date map of chronic exposure to PM_2.5_. The exposure map can provide crucial information for improved risk assessment and be used to improve our ability to study associations between long-term exposure to air pollution and adverse health effects in the United States.

## Supplemental Material

(258 KB) PDF
