# Supplementary material for: Comparison of Geostatistical Interpolation and Remote Sensing Techniques for Estimating Long-Term Exposure to Ambient PM2.5 Concentrations across the Continental United States
Source: Environ Health Perspect. 2012 Oct 2;120(12):1727–32. doi: 10.1289/ehp.1205006 (PMC3546366; doi:10.1289/ehp.1205006)
Supplement: (258 KB) PDF [file ehp.1205006.s001.pdf]

Supplemental Material

Comparison of Geostatistical Interpolation and Remote Sensing Techniques for Estimating Long-Term Exposure to Ambient $PM_{2.5}$ Concentrations across the Continental United States

Seung-Jae Lee[1], Marc L. Serre[2], Aaron van Donkelaar[3], Randall V. Martin[3,4], Richard T. Burnett[5], Michael Jerrett[6]

[1] Geospatial Development Department, Risk Management Solutions, Inc., Newark, California, USA

[2] Department of Environmental Sciences and Engineering, School of Public Health, University of North Carolina at Chapel Hill, Chapel Hill, North Carolina, USA

[3] Department of Physics and Atmospheric Science, Dalhousie University, Halifax, Nova Scotia, Canada

[4] The Harvard-Smithsonian Center for Astrophysics, Cambridge, Massachusetts, USA

[5] Population Studies Division, Health Canada, Ottawa, Ontario, Canada

[6] Division of Environmental Health Sciences, School of Public Health, University of California, Berkeley, California, USA

Geostatistical approaches may also suffer from the misuse of smoothing filters especially when a study domain spreads throughout broad spatial scales (i.e., U.S. continent). For example, there may exist a problem fitting the different periodicity in time on the west and east portion of the U.S. because $PM_{2.5}$ levels vary inversely by season with the highest levels being observed in the east during the summer months and the highest in the west during winter months (Bell et al. 2007 and also Figure 2A in the main text).

To show Kriging with the CSTM (KC) is more accurate than Kriging with SSTM (KS) we implemented cross-validation procedure: With the measurements at the space/time points $\boldsymbol{p}_{\mathrm{cv}}=[\boldsymbol{s}_{\mathrm{cv}},t_{\mathrm{cv}}]$ where $\boldsymbol{s}_{\mathrm{cv}}$ = randomly selected 25 sites across the U.S. (out of the locations in Figure 2A in the main text) and t_{cv} = each month in between 2001 and 2006, 1) we remove one measurement at a time, 2) re-estimate it using only nearby measurements, 3) iterate this kind of estimation procedure for all of the measurements at the 1800 $\boldsymbol{p}_{\mathrm{cv}}$ (25 sites × 72 months), and 4) compute estimation errors (difference between estimates and measurements left out of the cross-validation procedure). In the end we calculate Mean Square Error (MSE) (average of the squares of the estimation errors) as an indication of mapping accuracy for KS and KC. We test whether KC is more accurate than KS (equivalently the CSTM works better than the SSTM), as demonstrated below.

KC uses the covariance information (Eq. S1 and Figure S1) to obtain the kriging weight in Eq. [2] in the main text and calculates the mean trend values $\boldsymbol{m}_X(\boldsymbol{p}_{\mathrm{d}})$ at data points and $\boldsymbol{m}_X(\boldsymbol{p}_{\mathrm{cv}})$ at the cross-validation points (1800 spatiotemporal points). The MSE of KC is only 0.0561 (log-$\mu g/m^3)^2$ whereas that of KS (its covariance was not shown here) is 0.0635 (log-$\mu g/m^3)^2$. The MSE change from the latter to the former -11.65% indicating the former is more accurate than the latter by 11.65%. KC hardly outperforms KS at certain cross-validation points over space and time (thus only the 11.65% improvement overall) where there are no nearby measurements. However KC is still more accurate than KS and it is the interpolation method to contrast with the $PM_{2.5}$ estimates using remote sensing (i.e., referred to as RS in the main text).

With CSTM-induced residuals ($PM_{2.5}$ measurements − CSTM) we may estimate space/time variability (experimental covariance, also see the circles in Figure S1) for a given spatial lag r and temporal lag τ. The covariance may be parameterized by sill ($v_{01} - v_{04}$) and range ($a_{r2} - a_{r4}$, $a_{t2} - a_{t4}$) in a covariance model (red curve in Figure S1) that fits the experimental covariance, i.e.:

$$
\begin{aligned}
c_{X_R}(r,\tau) &= v_{01}\delta(r)\delta(\tau) + v_{02}\exp\left(\frac{-3r}{a_{\mathrm{r}2}}\right)\exp\left(\frac{-3\tau}{a_{\mathrm{t}2}}\right) \\
&+ v_{03}\exp\left(\frac{-3r^2}{a_{\mathrm{r}3}}\right)\exp\left(\frac{-3\tau}{a_{\mathrm{t}3}}\right) + v_{04}\exp\left(\frac{-3r}{a_{\mathrm{r}4}}\right)\cos\left(\frac{2\pi\tau}{a_{\mathrm{t}4}}\right)
\end{aligned}
\qquad [S1]
$$

where v_{01}=0.62 (log-μg/m^3)^2, v_{02}=0.023 (log-μg/m^3)^2, v_{03}=0.0267 (log-μg/m^3)^2, v_{04}=0.0109 (log-μg/m^3)^2, $\delta(r)$=1 if r=0, $\delta(r)$=0 if r>0, $\delta(\tau)$=1 if τ =0, $\delta(\tau)$=1.3 if τ>0, $a_{\mathrm{r}2}$=20 km, $a_{\mathrm{t}2}$=1 month, $a_{\mathrm{r}3}$=1300 km, $a_{\mathrm{t}3}$=2 months, $a_{\mathrm{r}4}$=9000 km, and $a_{\mathrm{t}4}$=12 months. The covariance model (Eq. S1) is used for obtaining $\boldsymbol{c}_{k,d}$ and $\boldsymbol{c}_{k,d}$ in Eq. [2] in the main text which is an input for the KC estimator in Eq. [1] in the main text. The top and bottom plots in Figure S1 denote purely spatial (when temporal lag τ=0) and purely temporal (when spatial lag r=0) covariances, respectively. The spatial piece (top plot) is a linear combination of the nugget effect, exponential, and gaussian functions in *BMElib* (unc.edu/depts/case/BMELIB), whereas the temporal portion (bottom plot) includes a linear combination of nugget effect, vertical shift, exponential function together with cosinusoidal functions associated with the seasonal effects of the $PM_{2.5}$ attribute.

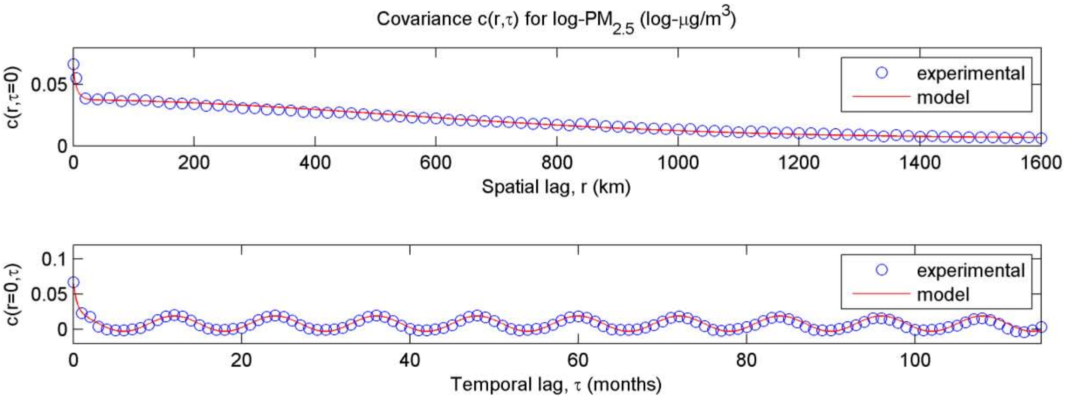

Supplemental Material, Figure S1: Space/time experimental covariance values and their covariance models using the CSTM-induced residuals

References

Bell ML, Dominici F, Ebisu K, Zeger SL, Samet M. 2007. Spatial and temporal variation in $PM_{2.5}$ chemical composition in the United States for health effects studies. Environ Health Persp 15(7):989-995.
